# Polygenic risk score-based phenome-wide association for glaucoma and its impact on disease susceptibility in two large biobanks

**DOI:** 10.1186/s12967-024-05152-4

**Published:** 2024-04-15

**Authors:** Jae-Seung Yun, Sang-Hyuk Jung, Su-Nam Lee, Seung Min Jung, Penn Medicine Biobank, Daniel J. Rader, Marylyn D. Ritchie, JoEllen Weaver, Nawar Naseer, Giorgio Sirugo, Afiya Poindexter, Yi-An Ko, Kyle P. Nerz, Meghan Livingstone, Fred Vadivieso, Stephanie DerOhannessian, Teo Tran, Julia Stephanowski, Salma Santos, Ned Haubein, Joseph Dunn, Anurag Verma, Colleen Morse Kripke, Marjorie Risman, Renae Judy, Colin Wollack, Anurag Verma, Shefali S. Verma, Scott Damrauer, Yuki Bradford, Scott Dudek, Theodore Drivas, Hong-Hee Won, Dokyoon Kim, Jin A. Choi

**Affiliations:** 1 Department of Internal Medicine, St. Vincent’s Hospital, College of Medicine, The Catholic University of Korea, Seoul, Republic of Korea; 2 Department of Biostatistics, Epidemiology and Informatics, Perelman School of Medicine, University of Pennsylvania, Philadelphia, PA USA; 3 Samsung Advanced Institute for Health Sciences and Technology (SAIHST), Samsung Medical Center, Sungkyunkwan University, Seoul, Republic of Korea; 4 Samsung Genome Institute, Samsung Medical Center, Seoul, Republic of Korea; 5 Institute for Biomedical Informatics, University of Pennsylvania, Philadelphia, PA USA; 6 Department of Ophthalmology, College of Medicine, St. Vincent’s Hospital, College of Medicine, The Catholic University of Korea, Seoul, Republic of Korea

## Abstract

**Background:**

Glaucoma is a leading cause of worldwide irreversible blindness. Considerable uncertainty remains regarding the association between a variety of phenotypes and the genetic risk of glaucoma, as well as the impact they exert on the glaucoma development.

**Methods:**

We investigated the associations of genetic liability for primary open angle glaucoma (POAG) with a wide range of potential risk factors and to assess its impact on the risk of incident glaucoma. The phenome-wide association study (PheWAS) approach was applied to determine the association of POAG polygenic risk score (PRS) with a wide range of phenotypes in 377, 852 participants from the UK Biobank study and 43,623 participants from the Penn Medicine Biobank study, all of European ancestry. Participants were stratified into four risk tiers: low, intermediate, high, and very high-risk. Cox proportional hazard models assessed the relationship of POAG PRS and ocular factors with new glaucoma events.

**Results:**

In both discovery and replication set in the PheWAS, a higher genetic predisposition to POAG was specifically correlated with ocular disease phenotypes. The POAG PRS exhibited correlations with low corneal hysteresis, refractive error, and ocular hypertension, demonstrating a strong association with the onset of glaucoma. Individuals carrying a high genetic burden exhibited a 9.20-fold, 11.88-fold, and 28.85-fold increase in glaucoma incidence when associated with low corneal hysteresis, high myopia, and elevated intraocular pressure, respectively.

**Conclusion:**

Genetic susceptibility to POAG primarily influences ocular conditions, with limited systemic associations. Notably, the baseline polygenic risk for POAG robustly associates with new glaucoma events, revealing a large combined effect of genetic and ocular risk factors on glaucoma incidents.

**Information:**

The online version contains supplementary material available at 10.1186/s12967-024-05152-4.

## Introduction

Glaucoma is a leading cause of irreversible vision loss worldwide [1]. Adult-onset primary open-angle glaucoma (POAG), the major type of glaucoma, has an inheritable element but also results from a complex interplay among various environmental, genetic, and lifestyle factors [2]. Recent studies on the heritability of POAG suggest that genetic factors make a significant contribution to POAG, with high heritability [3, 4]. Similarly, a systemic meta-analysis reported high heritability in the individual endophenotypes responsible for POAG [5].

Genetic factors are mediated by intermediate phenotypes; these phenotype attributes known to contribute to risk for POAG can be classified as either ocular or systemic [6]. Well-recognized systemic risk factors include diabetes, hypertension, low diastolic blood pressure, migraine, and thyroid disease [7]. Among ocular risk factors, the most important is elevated intraocular pressure (IOP) [5]. However, given the fact that a significant proportion of patients diagnosed under the broad classification of open-angle glaucoma actually display IOP values within the normal range, it is supposed that individual biomechanical architecture around the optic nerve head could mediate susceptibility to glaucomatous damage. In particular, myopia, which involves elongation of the eye and resultant stretching of the peripapillary sclera and optic nerve, shows substantial causal association with POAG [8]. Corneal hysteresis (CH), a measure of viscoelastic damping of cornea, represents an alteration of ocular biomechanics. Low level of CH is associated with the development and progression of glaucoma [9–11]. The close association of myopia and CH with POAG suggests a role of altered ocular biomechanics in glaucoma pathogenesis [12].

Several large-scale genome-wide association studies (GWAS) on POAG have been conducted to date, identifying hundreds of associated loci [13]. Polygenic risk scores (PRS) are widely used to synthesize disease-associated single-nucleotide polymorphisms (SNPs) into a single score and offer a means of risk stratification for common chronic diseases, such as POAG [14, 15]. The polygenic nature of POAG suggests that a synthesizing measure such as a PRS could be useful for determining individual disease risk [15]. Previous research has shown that polygenic variants for POAG are associated with more than 2.5-fold increased odds of incident glaucoma, which is comparable to known monogenic risk variants, and they are ~ 15 times more prevalent than a monogenic variant in the general population [16]. A PRS for POAG has likewise demonstrated utility in risk stratification, with significant association with disease onset, disease severity and treatment intensity [14].

Considering that glaucoma is phenotypically linked with many systemic and ocular conditions, it is crucial to explore the associations between genetic predisposition to glaucoma and these phenotypes. A phenome-wide association study (PheWAS) is a hypothesis-free approach that tests a set of selected genetic variants or a PRS for association with hundreds of phenotypes without prior assumptions. As such, a PheWAS can be useful for investigating the genetic basis of glaucoma, where the pathogenesis and underlying genetic factors are not well understood.

In this study, we used genomic and phenotypic data from the UK Biobank and Penn Medicine Biobank to conduct a PRS-based PheWAS for POAG, exploring a wide range of disease phenotypes. We then assessed the distribution of patient characteristics across PRS-based glaucoma genetic risk groups and the associations of POAG PRS with ocular factors, focusing specifically on IOP, myopia, and CH. Finally, we investigated the impact of POAG PRS on risk of developing incident glaucoma and explored the combined impact of genetic factors and ocular factors on the risk of incident glaucoma.

## Materials and methods

### Study design

Our analysis was conducted in two steps. First, we performed a PheWAS of POAG PRS with all available disease phenotypes in the UK Biobank (discovery set) and in the Penn Medicine Biobank (replication set). Second, we focused on the association of the POAG PRS with ocular factors and incident glaucoma, using a prospective study design within the UK Biobank.

### Genotyping, quality control, and imputation

Genotyping and quality control (QC) procedures and imputation followed the standard practices and were performed per cohort-genotyping platform pair. Further details are described in Additional file 1: Method S1.

#### UK Biobank

The UK Biobank is a prospective cohort study comprising ~ 500,000 middle-aged UK residents aged 40–69 years, recruited from 2006 to 2010, who were followed up with on their health-related outcomes [17]. All participants gave written informed consent for the linkage of the UK Biobank dataset to their health-related records, including primary care records, hospital inpatient records, and cancer and death registry data [18]. The UK Biobank obtained initial ethical approval from the North West Multi-Centre Research Ethics Committee on June 17, 2011 (Ref 11/NW/0382). After this, the approval underwent renewal processes in 2016 (Ref 16/NW/0274) and 2021 (Ref 21/NW/0157), with the next scheduled renewal set for the year 2026. The present research was conducted using the UK Biobank Resource under application number 90981. All research adhered to the tenets of the Declaration of Helsinki.

The UK Biobank samples (version 3; March 2018) were genotyped using either the Affymetrix UK BiLEVE Axiom array or the Affymetrix UK Biobank Axiom array. Imputation via IMPUTE2 was conducted by UK Biobank researchers using the merged 1000 Genomes Project panel and UK 10K panel [19]. After imputation and QC, 377, 852 white-British participants were determined eligible for the genetic analyses.

#### Penn Medicine Biobank

The Penn Medicine Biobank was approved under IRB protocol #813913 and supported by the Perelman School of Medicine at University of Pennsylvania, a gift from the Smilow family, and the National Center for Advancing Translational Sciences of the National Institutes of Health under CTSA award number UL1TR001878.

The Penn Medicine Biobank consists of 43,623 unique samples that underwent genotyping with the GSA genotyping array (Illumina, SD, USA). we performed genotype imputation using Eagle2 [20] and Minimac4 [21] software on the TOPMed Imputation Server [22]. After imputation and QC, 27,933 white-European participants were deemed eligible for the replication analyses.

### PRS generation for POAG

To quantify genetic risk for glaucoma, we generated a PRS using summary statistics from the GWAS conducted by the International Glaucoma Genetics Consortium (IGGC) [13], which consisted of 12,713,176 variants. The GWAS was conducted with a fixed-effects meta-analysis of 15,229 POAG cases and 177,473 controls of European descent excluding UK Biobank samples and the summary statistics are available via GWAS Catalog [23], under the study accession identifier GCST90011767.

The PRS was constructed using PRS-CS (version 1.0.0) [24], which is a Bayesian polygenic method that infers the posterior mean effect size of each variant based on GWAS summary statistics and the linkage disequilibrium reference panel. The individual PRSs were computed from beta coefficients as the weighted sum of the risk alleles by applying PLINK version 1.90 [25]. Finally, 1,116,933 SNPs that overlapped with the HapMap3 (1,287,078 SNPs) database were utilized for the POAG PRS.

### Phenome-wide association study

The “*PheWAS*” R package (version 0.99.5.5) was used to perform PheWAS analyses [26]. In these analyses, POAG PRS was set as the independent variable, and disease phenotypes as dependent variables, with age, sex, genotyping array, and first 10 genetic principal components (PCs) of ancestry as covariates. Disease diagnosis category phenotypes were obtained by mapping the International Classification of Diseases, Ninth or Tenth Revision (ICD-9 and -10) diagnosis codes of the UK Biobank to 1,618 hierarchical phenotypes (PheCodes) categorized into 17 disease categories [26, 27]. We removed phenotypic codes with less than 100 cases and those concerning symptoms, injuries, and poisoning, resulting in 776 phenotypes in 15 disease categories that were included in our analysis. Of these, 767 were eligible for replication analysis in the Penn Medicine Biobank. The number of cases for each phenotype and detailed demographic information in each cohort are summarized in Additional file 1: Table S1. To account for multiple testing, we used a Bonferroni threshold of *P* < 0.0000644 (0.05/776) when determining significance among the results of our main analyses in the UK Biobank and *P* < 0.0000652 (0.05/767) for the replication analyses. Next, to obtain insight into comorbid conditions associated with the POAG phenotype, we conducted additional PheWAS analyses using a diagnosis of POAG as the independent variable.

### Inclusion and exclusion criteria for the prospective study within the UK Biobank

Out of the initial cohort of 502,409 UK Biobank participants across six assessment centers in the United Kingdom, a total of 377,852 participants were available in the data release following thorough QC. Within this subset, cases with accessible ophthalmic data, including spherical power, cylinder power, CH, corneal resistance factor, and IOP were identified and included (n = 83,804). To avoid the effect of surgery on refractive error, IOP, and CH, participants were excluded if they reported having received glaucoma surgery, cornea refractive surgery, cataract surgery, or if they had previous eye injury in either eye (n = 3455). Finally, cases with baseline glaucoma diagnosis were excluded from the analysis (n = 6801). Consequently, the study focused on a refined cohort of 73,548 participants for the investigation of the association between the POAG PRS and the occurrence of incident glaucoma. The flowchart showing participants included for analysis is shown in Additional file 1: Fig. S1.

### Measurement of variables

Medical history of hypertension, diabetes, and dyslipidemia at baseline was based on the self-report collected in an in-person interview at enrollment or on diagnostic and procedure codes in electronic health records. The definitions of baseline dyslipidemia, hypertension, and type 2 diabetes are described in Additional file 1: Table S2.

All participants who had available spherical equivalent (SE), Goldmann-correlated IOP (IOP_g_), and CH were used for this analysis. Participants underwent a detailed ophthalmologic examination in which non-cycloplegic refraction status was determined using an autorefractor (Tomey, Nagoya, Japan). The SE of refractive error was defined as the sphere component plus half the cylinder component. After measuring visual acuity and refraction, CH, corneal resistance factor, and IOP_g_ were measured with the Reichert Ocular Response Analyzer (ORA, Reichert, Inc., Depew, NY, USA). The average of the ORA pressure values was calibrated against Goldmann applanation tonometer measures to derive IOP_g_. Participants who had eye surgery within the previous 4 weeks or those with possible eye infections were precluded from the determination of IOP. Measurements were excluded from analysis if the participants had a history of corneal surgery, refractive surgery, or injuries in either eye; additionally, participants with a history of glaucoma surgery were excluded and left eyes with missing data were excluded.

Participants were identified as having glaucoma if they self-reported glaucoma on the eye problems/disorders (UK Biobank data field: 6148) or non-cancer illness (UK Biobank data field: 20002), or had an ICD-9/10 diagnosis code for POAG, other glaucoma, or glaucoma, unspecified, based on the use of these codes in prior in population-based and registry-based studies of genetic risk for glaucoma [28, 29]. (Additional file 1: Table S2).

### Statistical analyses

In the prospective study conducted within the UK Biobank, measures from the left eye were used as the outcome variable, consistent with previous reports [30, 31]. In the UK Biobank study, the measurement of the left eye was done, as per the study protocol, following the collection of right eye data. This sequencing choice suggests that left eye data in our cohort may be less susceptible to artifacts, such as blinking. Reference was made to previous authoritative articles that reported IOP and CH in the UK Biobank, where analyses were based on left-eye data. Continuous variables are reported as means with standard deviations (SD) and categorical variables as frequencies and proportions. In some analyses, individuals were classified according to the degree of myopia. Myopia overall was defined as having a SE of ≤ − 0.5D and mild myopia, moderate myopia, and high myopia as − 3.0D < SE ≤ − 0.5D, − 6.0D < SE ≤ − 3.0D, and SE ≤ − 6.0D, respectively. Regarding CH, participants with a value of ≤ 10.1 mmHg were considered to have low CH; this cutoff was determined to be significantly associated with glaucoma prevalence in a previous study based on UK Biobank data [31]. Those with a value > 10.1 mmHg were classified as having high CH. In terms of IOP, individuals were categorized into two groups: those with ocular hypertension (IOP_g_ ≥ 21 mmHg) and those without (IOP_g_ < 21 mmHg).

**Fig. 1:**
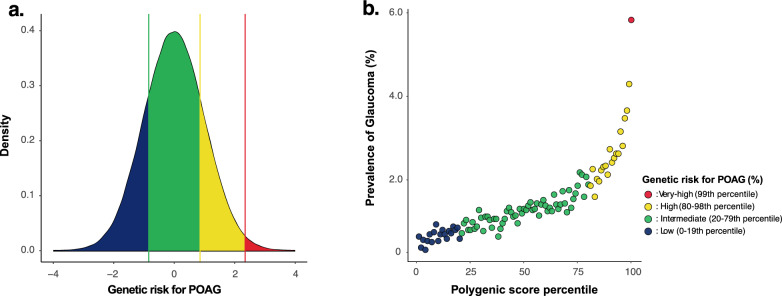
Density (**A**) and prevalence plot (**B**) according to the PRS distribution for POAG (n = 377, 852)

As done in previous studies, we categorized study participants based on the PRS into low, intermediate, or high genetic risk groups [32, 33], and also further classified the top 1% of the PRS distribution as a very-high-risk group in light of the curve of cumulative incidence of disease prevalence over the PRS distribution (Fig. 1). Thus, participants were categorized into the following four risk subgroups: low, 0–19th percentile; intermediate, 20–79th percentile; high; 80–98th percentile; and very high, 99th percentile.

The baseline clinical characteristics of participants were compared using analysis of variance (ANOVA) for continuous variables and the Chi-square test for categorical variables. To evaluate the association of glaucoma genetic risk with SE, IOP_g_, and CH, we applied multivariable logistic and linear regression analyses after adjusting for age, sex, genotyping array, and the first 10 PCs of ancestry, and other factors determined on the basis of previous studies [30, 31]. We performed a Cox proportional hazards regression analysis, utilizing genetic risk and ocular factors, with age at glaucoma onset or age at the last clinical visit as time variables and the diagnosis of glaucoma as a status and calculated hazard ratio (HR). Kaplan–Meier survival curves and standardized cumulative incidence according to categories of PRS for POAG were plotted to assess potential disparities in survival among genetic risk groups. Subsequently, we conducted joint association analyses to investigate the interplay between genetic and ocular factors on the development of incident glaucoma. Statistical tests were two-sided, and *P* < 0.05 was considered statistically significant. All statistical analyses were conducted using R version 3.9.0.

## Results

### PheWAS

**Fig. 2:**
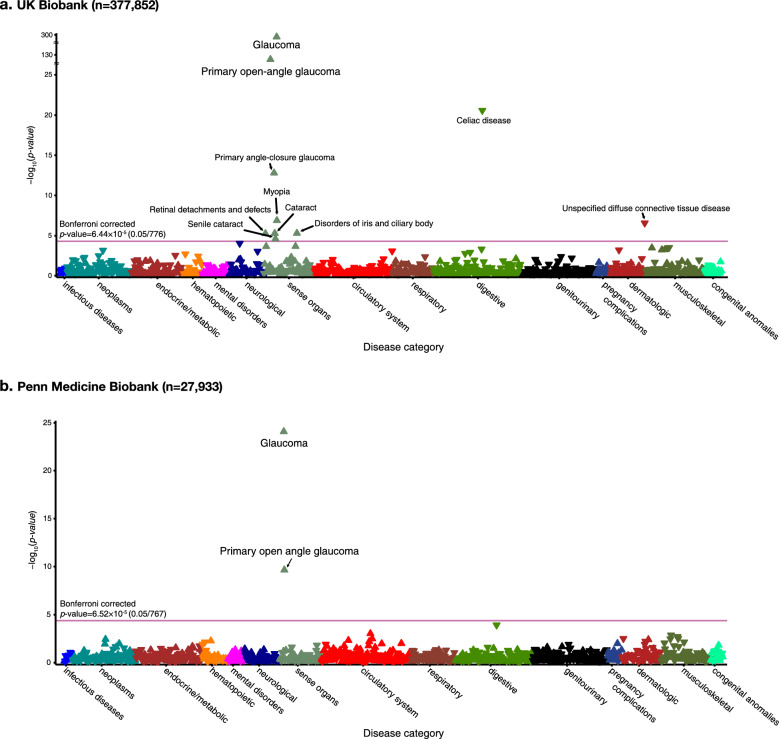
Significance plot for all phenotypes for POAG PRS, grouped by disease categories from discovery set and replication set. **A** Significance plot for all phenotypes for POAG PRS, grouped by disease categories from UK Biobank. PheCodes are organized and plotted by disease category on the x-axis, and the y-axis represents the − log10 of uncorrected *P* values of two-sided test for linear regression between POAG PRS and each of the phenotype. Each point represents a single PheCode, and the color indicates their corresponding categories. The horizontal line is marked at the Bonferroni threshold of significance for multiple testing (*P* < 5.21 × 10^−5^). The representative significant associations in each category are annotated in the figure. The direction of each arrowhead corresponds to increased risk (up) or decreased risk (down). The discovery samples consist of 377,852 participants in the UK Biobank and the exact sample size for each phenotype are presented in Additional file 2. **B** Significance plot for all phenotypes for POAG-PRS, grouped by disease categories from Penn Medicine Biobank (replication sample). PheCodes are organized and plotted by category on the x-axis, and the y-axis represents the − log10 of uncorrected *P* values of two-sided test for linear regression between POAG PRS and each of the phenotype. Each point represents a single PheCode, and the color indicates their corresponding categories. The horizontal red line is marked at the Bonferroni threshold of significance for multiple testing (*P* < 6.52 × 10^−5^). The representative significant associations in each category are annotated in the figure. The direction of each arrowhead corresponds to increased risk (up) or decreased risk (down). The replication samples consist of 27,933 participants and the exact sample size for each phenotype is presented in Additional file 2 .

The UK Biobank (discovery set) sample comprised 53.7% female (mean age: 56.9 ± 7.8) individuals of white-British ancestry, and the Penn Medicine Biobank (replication set) sample included 45.2% female (mean age: 57.4 ± 16.2) of white-European ancestry. In the PheWAS analyses, higher genetic predisposition to POAG was exclusively associated with ocular disease phenotype. In the discovery set, we observed significant associations between POAG PRS and 10 disease phenotypes, which remained significant after Bonferroni corrections; these phenotypes were glaucoma, POAG, celiac disease, primary angle-closure glaucoma, myopia, unspecified diffuse connective tissue disease, disorders of iris and ciliary body, cataract, retinal detachments, and senile cataract. Disease phenotypes categorized as involving sensory organs, which includes eye-related disorders, displayed positive associations with POAG PRS, while celiac disease and unspecified diffuse connective tissue disease showed negative associations (Fig. 2A). Of the 10 disease phenotypes that exhibited significant associations in the discovery set, eight demonstrated the same direction of effect in the replication set; however, only two, namely glaucoma and POAG, remained significant after Bonferroni’s correction (Fig. 2B). A summary of the 776 disease phenotypes included in the PheWAS and their odds ratios (ORs) are presented in Additional file 2: Table S3. In contrast, when we conducted PheWAS analyses with a diagnosis of POAG as the independent variable, we identified a notable overrepresentation of multiple systemic disease phenotypes, across the spectrum of disease categories (Additional file 1: Fig. S2 and Additional file 2: Table S4).

### The association of POAG PRS with ocular factors

Demographic baseline information for all 73,548 participants and systemic and ocular characteristics for each PRS group are presented in Table 1. Overall, the prevalence of systemic diseases, including diabetes, hypertension, and dyslipidemia, and the proportion of current smokers and alcohol consumers, were not significantly different across the POAG genetic risk groups. Participants at higher genetic risk for glaucoma tended to have a lower body mass index (BMI) than those at low genetic risk (*P* < 0.001). Importantly, there were distinctive differences in the ocular characteristics across the genetic risk groups, with the trend showing that higher genetic risk accompanied higher IOP, lower SE, and lower CH (all *P* < 0.001). Compared with the low genetic risk group, the very high-risk category was associated with high IOP in multivariable logistic regression analyses (OR, 3.84; 95% confidence interval (CI) 2.98–4.89), myopia (OR, 1.28; 95% CI 1.06–1.52) and low CH (OR, 1.27; 95% CI 1.06–1.51). (Table 2; Results of multivariable linear regression analyses are shown in Additional file 1: Table S5).

**Table 1:** Summary of population characteristics across POAG genetic risk groups in UK Biobank data (n = 73,548)

POAG genetic risk group	Total	Low risk	Intermediate risk	High risk	Very high risk	*P* value
	(N = 73,548)	(N = 14,571)	(N = 44,413)	(N = 13,856)	(N = 708)	
Age (years)	56.9 ± 7.8	57.1 ± 7.8	56.9 ± 7.8	56.9 ± 7.8	57.1 ± 7.9	0.191
Sex (%)						0.335
Female	39,342 (53.5)	7796 (53.5)	23,660 (53.3)	7501 (54.1)	385 (54.4)	
Male	34,206 (46.5)	6775 (46.5)	20,753 (46.7)	6355 (45.9)	323 (45.6)	
Current smoker (%)	6912 (9.4)	1369 (9.4)	4233 (9.5)	1253 (9.0)	57 (8.1)	0.214
Alcohol consumption (%)						0.979
Never	12,674 (17.2)	2503 (17.2)	7678 (17.3)	2372 (17.1)	121 (17.1)	
< 2 times per week	27,062 (36.8)	5310 (36.5)	16,351 (36.8)	5142 (37.1)	259 (36.7)	
< 3–4 times per week	17,602 (23.9)	3525 (24.2)	10,622 (23.9)	3279 (23.7)	176 (24.9)	
Daily	16,168 (22.0)	3222 (22.1)	9742 (21.9)	3054 (22.1)	150 (21.2)	
Townsend deprivation index	− 1.3 ± 2.8	− 1.4 ± 2.8	− 1.3 ± 2.8	− 1.3 ± 2.8	− 1.2 ± 2.8	0.461
Systemic parameters						
Hypertension (%)	20,208 (27.5)	4040 (27.7)	12,200 (27.5)	3783 (27.3)	185 (26.1)	0.726
Diabetes (%)	14,201 (19.3)	2879 (19.8)	8616 (19.4)	2593 (18.7)	113 (16.0)	0.016
Dyslipidemia (%)	3032 (4.4)	615 (4.5)	1805 (4.4)	589 (4.6)	23 (3.5)	0.409
Obesity (%)	17,549 (23.9)	3604 (24.8)	10,620 (24.0)	3163 (22.9)	162 (22.9)	0.002
Systolic BP (mmHg)	140.6 ± 19.5	140.8 ± 19.7	140.5 ± 19.5	140.4 ± 19.6	140.8 ± 19.0	0.349
Diastolic BP (mmHg)	82.0 ± 10.6	82.0 ± 10.6	82.0 ± 10.6	82.0 ± 10.5	82.2 ± 10.2	0.873
HbA1c (%)	5.4 ± 0.6	5.4 ± 0.6	5.4 ± 0.6	5.4 ± 0.6	5.4 ± 0.6	0.745
LDL cholesterol (mg/dL)	137.3 ± 33.4	137.4 ± 33.6	137.5 ± 33.4	136.8 ± 33.3	137.1 ± 32.4	0.217
BMI (kg/m^2^)	27.4 ± 4.7	27.4 ± 4.8	27.4 ± 4.7	27.2 ± 4.7	27.1 ± 4.6	0.001
Ocular parameters						
Spherical power (D)	− 0.7 ± 2.8	− 0.5 ± 2.8	− 0.7 ± 2.8	− 0.8 ± 2.9	− 1.1 ± 3.0	< 0.001
Cylinder power (D)	− 0.8 ± 3.0	− 0.6 ± 2.9	− 0.8 ± 3.0	− 1.0 ± 2.8	− 1.1 ± 3.4	0.095
Spherical equivalent (D)	− 0.2 ± 2.8	− 0.1 ± 2.7	− 0.2 ± 2.8	− 0.4 ± 2.8	− 0.6 ± 2.9	< 0.001
IOP_g_ (mmHg)	15.8 ± 3.9	15.1 ± 3.7	15.8 ± 3.9	16.6 ± 4.1	17.5 ± 4.5	< 0.001
Corneal hysteresis (mmHg)	10.7 ± 2.5	10.8 ± 2.3	10.7 ± 2.5	10.6 ± 2.6	10.3 ± 2.3	< 0.001

Data are n (%) or mean (SD)

*POAG* primary open angle glaucoma, *PRS* polygenic risk score, *BMI* body mass index, *BP* blood pressure, *LDL* low-density lipoprotein, *ACR* albumin-creatinine ratio, *D* diopter, *IOP*_*g*_ Goldmann-correlated intraocular pressure, *SD* standard deviation

**Table 2:** Multivariable logistic regression analyses of POAG genetic risk for baseline intraocular pressure, refractive error, and corneal hysteresis (n = 73,548)

	High IOP				Myopia				Low CH			
	Model 1		Model 2		Model 1		Model 2		Model 1		Model 2	
	OR 95% CI	*P*	OR 95% CI	*P*	OR 95% CI	*P*	OR 95% CI	*P*	OR 95% CI	*P*	OR 95% CI	*P*
POAG PRS												
Low	Ref.		Ref.		Ref.		Ref.		Ref.		Ref.	
Intermediate	1.53 (1.41–1.67)	< 0.001	1.58 (1.44–1.74)	< 0.001	1.06 (1.02–1.10)	< 0.001	1.06 (1.01–1.16)	0.0131	1.09 (1.05–1.14)	< 0.001	1.07 (1.02–1.12)	< 0.001
High	2.37 (2.16–2.60)	< 0.001	2.38 (2.15–2.65)	< 0.001	1.19 (1.13–1.25)	< 0.001	1.18 (1.11–1.25)	< 0.001	1.23 (1.17–1.29)	< 0.001	1.21 (1.14–1.28)	< 0.001
Very high	3.93 (3.17–4.84)	< 0.001	3.84 (2.98–4.89)	< 0.001	1.35 (1.16–1.58)	< 0.001	1.28 (1.06–1.52)	0.0078	1.29 (1.10–1.50)	0.002	1.27 (1.06–1.51)	0.0103

*POAG* primary open angle glaucoma, *PRS* polygenic risk score, *OR* odds ratio, *CI* confidence interval

Myopia was defined as having a spherical equivalent of ≤ − 0.5D, low corneal hysteresis (CH) as having value of ≤ 10.1 mmHg, and high IOP (intraocular pressure) as having value of ≥ 21 mmHg

Model 1: Age + sex + genotyping array + first ten principal components of ancestry

Model 2: Model 1 + BMI + Income + Smoking + systolic blood pressure + diastolic blood pressure + LDL cholesterol + HbA1c + hypertension

### Association of POAG PRS with incident glaucoma

**Fig. 3:**
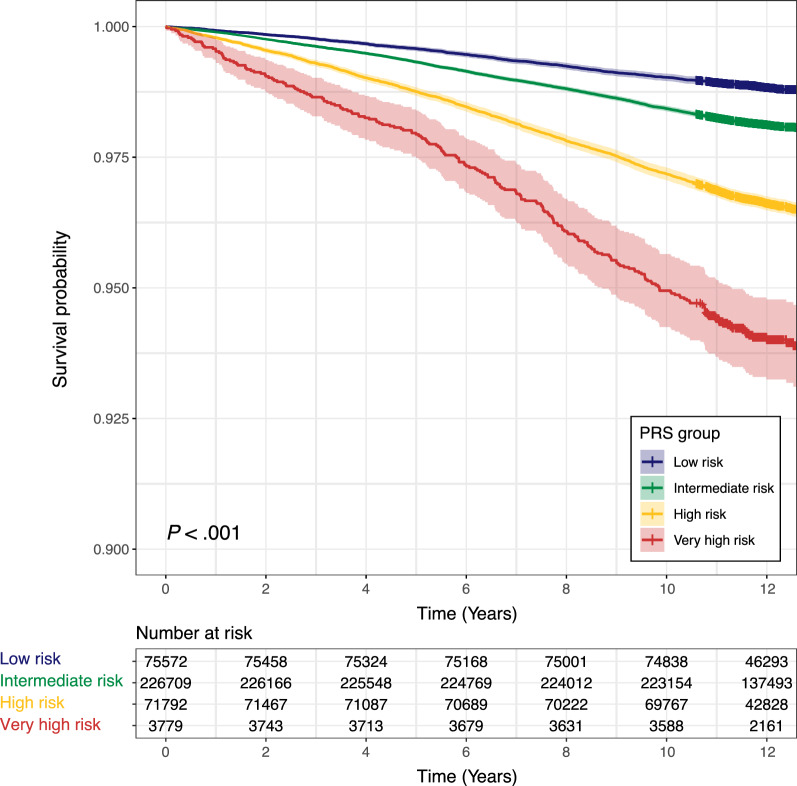
Kaplan–Meier survival curves according to categories of a polygenic risk score for primary open-angle glaucoma (POAG). The probability of survival over time for various genetic risk groups was significantly different (*P* < 0.001, Log-rank test)

The median duration of follow-up was 11.1 years, during which 1300 participants were identified as incident glaucoma cases. Compared with low genetic risk, higher genetic risk was associated with a higher risk of incident glaucoma during follow-up (*P* for trend < 0.001) (Table 3; Kaplan–Meier survival curves and cumulative incidences are shown in Fig. 3 and Additional file 1: Fig. S3 and S4). After adjusting for potential confounding factors, a 1-SD increase in POAG PRS was associated with 64% greater risk of incident glaucoma (HR, 1.64; 95% CI 1.54–1.75; *P* < 0.001). Notably, participants in the very high-risk group were 8.76 times more likely to have glaucoma than those in the low-risk group (Table 3).

**Table 3:** Hazard ratio for incident POAG according to POAG genetic risk (n = 73,548)

	No of events/total no. of participants	Incidence rate per 1000 person-year (95% CI)	Absolute risk (%)	Crude		Model 1		Model 2	
				HR (95% CI)	*P* value	HR (95% CI)	*P* value	HR (95% CI)	*P* value
POAG genetic risk									
Low	123/14,571	0.76 (0.63–0.90)	0.84	Ref.		Ref.		Ref.	
Intermediate	723/44,413	1.47 (1.36–1.58)	1.63	1.94 (1.60–2.34)	< 0.001	1.96 (1.62–2.37)	< 0.001	2.19 (1.74–2.77)	< 0.001
High	414/13,856	2.71 (2.46–2.98)	2.99	3.58 (2.93–4.38)	< 0.001	3.66 (2.99–4.47)	< 0.001	4.14 (3.25–5.29)	< 0.001
Very high	40/708	5.19 (3.71–7.06)	5.65	6.86 (4.80–9.80)	< 0.001	6.92 (4.84–9.89)	< 0.001	8.76 (5.87–13.08)	< 0.001
Per SD increase	1300/73,548	1.59 (1.51–1.68)	1.77	1.60 (1.52–1.69)	< 0.001	1.61 (1.52–1.70)	< 0.001	1.64 (1.54–1.75)	< 0.001

*POAG* primary open angle glaucoma, *PRS* polygenic risk score, *HR* hazard ratio, *CI* confidence interval, *SD* standard deviation

Model 1: Age + sex + genotyping array + first ten principal components of ancestry

Model 2: Model 1 + body mass index + smoking status + income status + systolic blood pressure + diastolic blood pressure + LDL cholesterol + HbA1c + Hypertension

### Association of ocular factors with incident glaucoma

In comparison to individuals exhibiting IOP < 21 mmHg, those with IOP ≥ 21 mmHg demonstrated an increased risk of incident glaucoma (HR, 6.74; 95% CI 5.89–7.71; *P* < 0.001). Notably, participants with moderate to high myopia showed an increased risk of glaucoma compared to those with non to mild myopia (HR, 1.59; 95% CI 1.36–1.88; *P* < 0.001). Furthermore, participants with CH ≤ 10.1 mmHg showed an increased risk of glaucoma compared to those with CH > 10.1 mmHg (HR, 1.24; 95% CI 1.09–1.42; *P* < 0.001) (Table 4 and results of continuous scale are shown in Additional file 1: Table S6).

**Table 4:** Hazard ratio for incident POAG according to the presence of ocular risk factors (n = 73,548)

	No of events/total no. of participants	Incidence rate per 1000 person-year (95% CI)	Absolute risk (%)	Crude		Model 1		Model 2	
				HR (95% CI)	*P* value	HR (95% CI)	*P* value	HR (95% CI)	*P* value
IOP									
< 21 mmHg	794/65,781	1.09 (1.01–1.16)	1.21	Ref.		Ref.		Ref.	
≥ 21 mmHg	462/5786	7.45 (6.78–8.16)	7.99	6.89 (6.14–7.72)	< 0.001	6.30 (5.61–7.07)	< 0.001	6.74 (5.89–7.71)	< 0.001
Myopia									
Non to mild	1069/63,708	1.51 (1.42–1.61)	1.68	Ref.		Ref.		Ref.	
Mod to high	1300/73,548	2.13 (1.86–2.42)	2.35	1.41 (1.22–1.62)	< 0.001	1.58 (1.37–1.82)	< 0.001	1.59 (1.36–1.88)	< 0.001
CH									
> 10.1 mmHg	647/43,703	1.33 (1.23–1.44)	1.48	Ref.		Ref.		Ref.	
≤ 10.1 mmHg	609/27,864	1.97 (1.82–2.14)	2.19	1.48 (1.33–1.65)	< 0.001	1.32 (1.18–1.48)	< 0.001	1.24 (1.09–1.42)	0.0009

*POAG* primary open angle glaucoma, *CH* corneal hysteresis, *IOP* intraocular pressure, *HR* hazard ratio, *CI* confidence interval

Model 1: Age + sex + genotyping array + first ten principal components of ancestry

Model 2: Model 1 + body mass index + smoking status + systolic blood pressure + diastolic blood pressure + LDL cholesterol + HbA1c + Hypertension + use of anti-glaucoma eyedrops

### Joint associations of POAG PRS with ocular factors

**Fig. 4:**
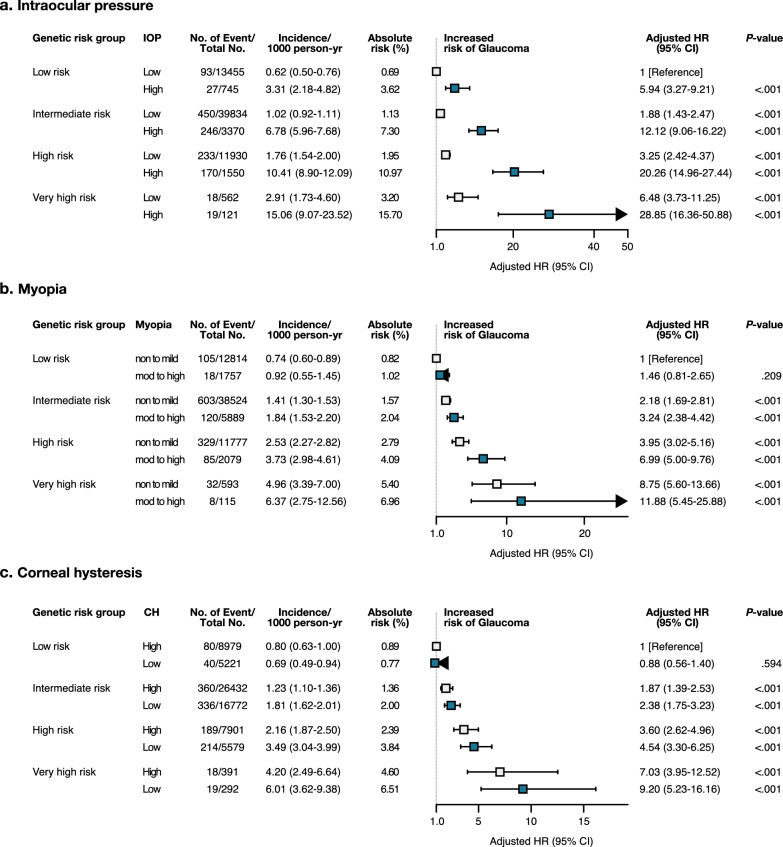
Forest plot of hazard ratio according to genetic risk and ocular risk factors (n = 73,548). Cox regression model was adjusted for age, sex, genotyping array, first 10 genetic principal components of ancestry, BMI, income, smoking status, systolic and diastolic blood pressure, low-density lipoprotein, HbA1c, and use of hypertensive medication. High intraocular pressure (IOP) was defined as having value of ≥ 21 mmHg, and myopia as having a spherical equivalent of ≤ -0.5D, and low corneal hysteresis (CH) as having value of ≤ 10.1 mmHg

To explore the effect of ocular risk factors on glaucoma risk according to genetic risk, we stratified the ocular factors by PRS category (Fig. 4). We observed a monotonic association between increasing PRSs and ocular factors and a higher risk of incident glaucoma. In particular, the participants with the very high-PRS and high IOP had the highest risk for incident glaucoma (HR, 28.85; 95% CI 16.36–50.88; *P* < 0.001). In addition, the participants with the very high-PRS and moderate-high myopia exhibited the substantial risk for incident glaucoma with HR of 11.88 (95% CI 5.45–25.88), and those with very high-PRS and low CH with HR of 9.20 (95% CI 5.23–16.16; *P* < 0.001).

## Discussion

Our study aimed to identify a broad spectrum of phenotypes associated with POAG genetic risk and to investigate how this genetic risk interacts with ocular risk factors in the development of POAG. Notably, we observed that individuals in the top 1% PRS for POAG exhibited a significantly higher risk of new glaucoma events, with a HR of 8.8. Furthermore, our analysis of the joint associations of polygenic risk and ocular risk factors revealed that individuals with low baseline IOP and very high polygenic risk had a similar hazard ratio for developing glaucoma compared to those with high baseline IOP and low polygenic risk (absolute risk, 3.62% vs. 3.20%).

Increased IOP is considered to be the most important evidence-based risk factor for the development and progression of glaucoma, with a considerable number of SNPs in the POAG PRS implicated in increasing IOP [6, 16]. Nevertheless, noteworthy is the observation that disease susceptibility was substantially increased even among individuals with a high genetic susceptibility but low IOP. This underscores the strong influence of polygenic risk on the development of glaucoma. In this regard, in addition to regular IOP checks, the assessment of POAG genetic risk could serve as a robust tool for the early identification of individuals at high risk for the disease.

Genetic predisposition to POAG has been previously associated with the severity and predictive aspects of the disease. Individuals in the top 5% PRS reportedly had 2.8-fold increased odds of glaucoma [16]. In the cohort study of PROGRESSA, subjects with early POAG with high polygenic risk had faster structural and functional progression [34]. In the glaucoma population, subjects with high PRS had increased risk of younger age at diagnosis and higher requirement for incisional surgery [14]. In addition to the findings of the previous studies, we also demonstrated the robust synergistic effect between genetic and ocular factors in predicting the future glaucoma development, showing that the participants with high genetic burden exhibited 28.9-fold, 11.8-fold, and 9.2-fold increased risk of glaucoma incidence, when they accompanied high IOP, moderate to high myopia, and low CH, respectively. Craig et al. [14] previously reported the improved predictive capacity through the integration of polygenic risk and IOP and vertical CD ratio when estimating glaucoma odds ratios. However, to our knowledge, a large and well-phenotyped prospective cohort study assessing the comprehensive joint association of ocular factors and genetic risk has not been reported. The manifestation of genetic risk for glaucoma substantially increased risk of glaucoma incidence by joint associations of genetic and ocular factors, emphasizing the significance of risk stratification based on genetic and ocular risk factors for early detection and prevention of the disease.

In this study, we conducted a PRS-based PheWAS analysis to gain better insight and identify associations between genetic predisposition to POAG and disease phenotypes available in the UK Biobank and Penn Medicine Biobank cohorts. Our findings consistently revealed that the genetic liability for POAG to have a predominant impact on the development of ocular conditions, with little correlation to systemic disease phenotypes in both the discovery and replication sets.

In contrast to the results of a PRS-based PheWAS, our investigation using PheWAS with POAG diagnosis as the independent variable revealed significant associations with various systemic conditions, which were previously identified as systemic risk factors for glaucoma in epidemiologic studies [7, 35–39]. Despite the distinct disease associations of POAG, we found no positive associations between genetic liability for POAG and systemic disease phenotypes. In accordance with our study, a previous work has reported limited genetic correlation of diabetes with glaucoma-related endophenotypes and POAG [40]. Kolli et al. [41] recently reported that the association between glaucoma and cardiometabolic disease differed by genetic risk for glaucoma, reporting that glaucoma patients with low genetic risk tended to have a higher prevalence of cardiometabolic disease, while the opposite trend was observed for those with high genetic risk. The differential PheWAS results of POAG and POAG polygenic risk in our study suggests that systemic conditions may have a substantial impact on the development of glaucoma, potentially by sharing confounding risk factors with POAG or serving as prominent non-genetic risk factors. 

In our study, both POAG PRS and POAG diagnosis were significantly associated with overrepresentation of the ocular disease phenotypes such as cataract, uveitis, retinal detachment, and myopia with the same directional effect. POAG is reportedly significantly associated with cataracts and myopia [42, 43]. Elevated IOP and the use of glaucoma medication increase the risk of 5-year incident cataracts [42]; additionally, there is a higher prevalence of retinal detachment among patients with glaucoma, particularly in cases where glaucoma is advanced or the IOP is poorly controlled [44]. Meanwhile, in consistent with results from a recent large multi-ethnic meta-analysis of GWASs, we found diseases such as celiac disease or unspecified diffuse connective tissue disease to have negative associations with POAG PRS, suggesting that these conditions may have genetic factors that are protective against the development of glaucoma or vice versa [45]. Predominant association of genetic predisposition to POAG with ocular disease phenotypes in the PheWAS suggest that common genetic variants associated with POAG primarily drive ocular conditions, rather than involving disease pathways associated with metabolic traits.

In line with the finding from the results of PheWAS, our prospective study confirmed that the initial presence of ocular factors, specifically elevated IOP, myopia, and low CH, was significant predictors for the development of incident glaucoma. Furthermore, these ocular factors were significantly associated with the genetic risk of glaucoma. Choquet et al. [46] reported that myopia and glaucoma demonstrate a shared genetic architecture. Recently, low CH was reportedly associated with POAG PRS and greater prevalence of disc hemorrhage [47]. These significant associations of POAG PRS with myopia and CH implicates that the genetic pathways responsible for POAG have clinically significant implications for the pathogenic role of the ocular biomechanics. In addition to ocular hypertension, these altered ocular biomechanics could collectively increase ocular susceptibility to glaucomatous damage in the general population. Further prospective studies are necessary to clarify the association between myopia, CH, and the onset of new glaucoma.

In this study, we developed and assessed PRS-PheWAS for individuals of European ancestry. Nonetheless, we acknowledge the importance of developing PRSs for groups of non-European ancestry, given the potential challenges associated with the transferability of PRSs across different ancestries and ethnic groups. Recently, Verma et al. [48] reported a multi-cohort GWAS in Africans including the Penn Medicine Biobank and showed that, for individuals of African descent, PRS for POAG based on African GWAS outperformed PRS from a much larger GWAS derived from European populations. Additionally, the study by Gharahkhani et al. [49] conducted a large multi-ethnic meta-analysis of GWAS, confirming that several genetic variants share their effects on POAG across ethnicities, including those of European descent. However, it also identified ancestry-specific genetic factors (e.g., rs16944405 in IQGAP1), indicating that polygenic diseases with numerous ancestry-specific genetic variants, like POAG, might exhibit lower transferability across ethnicities in PRS analyses. Therefore, it is important to perform GWAS and validate PRS and PRS-PheWAS results in multi-ethnic populations in further studies.

In this study, to maintain consistency, the PRS-CS approach was used in the PRS-PheWAS and analyses of joint associations with ocular factors. This is one of the widely used Bayesian polygenic modeling approaches and minimizes parameter selection bias through the auto option using the linkage disequilibrium reference panel. However, it is also crucial to acknowledge that several other approaches (PRS-CT [clumping and thresholding], PRSice-2, lassosum, LDpred2, SBLUP, SbayesR, DBSLMM, etc.) [50–55] exist, and different methods can influence the results. For transparency and reproducibility in genetic research, we used the PRS model (PRS-CS) that is publicly available.

This study has several strengths. First, to better identify the multiple disease phenotypes associated with POAG PRS, we used a hypothesis-free approach, which overcomes an incomplete understanding of the disease mechanism. Additionally, a notable strength of this study is the large sample size, which allowed us to investigate whether genetic markers associated with POAG may be related to ocular factors in the general population. Furthermore, our findings were overall replicated in an independent data set. The two cohort datasets used in this study might have distinct demographic and clinical characteristics. The UK Biobank is a prospective national cohort study based on healthy participants, whereas the Penn Medicine Biobank is an academic research cohort derived from a regional university hospital with diverse ancestries. Despite these potential differences, our replicated results might enhance the generalizability of our study, expanding its relevance to both the healthy population and those associated with a hospital setting. Next, we used a longitudinal study design to assess the impact of POAG PRS on glaucoma incidence. Finally, this study defined the very high-risk group as those with a top 1% PRS, as opposed to using deciles in previous studies [16, 41, 47]. This difference in risk stratification could potentially account for the elevated HR observed in this study.

However, the study also has limitations. First, the assessment of glaucoma progression was constrained by the limited parameters available within the datasets. Second, although we used POAG GWAS summary statistics from a large-scale meta-analysis, our analyses were limited to individuals of European ancestry. Therefore, further studies with other cohorts are warranted to verify the generalizability of our findings. Third, drawing upon the definition used in previous reports [28, 29], we defined POAG on the basis of ICD codes or self-reports. This method of definition could have resulted in overestimation or underestimation of disease. Fourth, our study is less likely to uncover POAG risk factors that have no or only a weak genetic link to the disease. The stringent statistical criteria applied in our PheWAS may have excluded associations that could be potentially noteworthy. Fifth, in our study, genotype data of the two biobanks were imputed using different imputation reference panels. To mitigate potential biases arising from these limitations, we focused our analysis on individuals of European descent and used the HapMap3 variants using the PRS-CS approach for PRS-PheWAS. Nevertheless, the accuracy and consistency of PRS could be further improved by using the same reference panel across discovery and replication datasets.

In conclusion, our study demonstrated the potential of PRS-based PheWAS in revealing associations between genetic risk factors for glaucoma and various ocular conditions. The findings emphasized the importance of considering genetic susceptibility in understanding glaucoma and highlighted shared genetic bases between glaucoma and other ocular conditions. We could find that the overall genetic liability to POAG primarily contributes to ocular conditions and has only limited associations with systemic disease phenotypes. Genetic predisposition to POAG accompanies lower CH, and refractive error, as well as ocular hypertension in the study population. The genetic risk for POAG demonstrated a robust association with the occurrence of incident glaucoma and the participants with high genetic burden exhibited a substantially increased risk of glaucoma incidence when they accompanied high IOP, moderate to high myopia, and low CH. The novel joint effect between genetic burden and ocular risk factors for POAG implicates that the interplay between genetic and ocular factors may substantially contribute to the increased risk of glaucoma, highlighting the necessity for rigorous glaucoma monitoring in populations with a high genetic burden.

### Supplementary Information

**Additional file 1:** **Acknowledgements.** Penn Medicine Biobank banner author list and contribution statements. **Method S1.** Detailed information on the genotype data quality control and imputation procedures. **Figure S1.** The flowchart showing participants included for analysis. **Figure S2.** Significance plot for all phenotypes for the diagnosis of primary open-angle glaucoma (POAG), grouped by disease categories. **Figure S3.** Log–log survival curves according to categories of polygenic risk score for primary open-angle glaucoma (POAG) (n = 73,548). **Figure S4.** Standardized cumulative incidence according to categories of polygenic risk score for primary open-angle glaucoma (POAG). **Table S1.** Descriptions of disease phenotypes included in the PheWAS analyses for POAG PRS. **Table S2.** Detailed definitions of glaucoma and comorbidities. **Table S3.** Demographic information for discovery and replication samples. **Table S4.** Descriptions of disease phenotypes included in the PheWAS analyses for the diagnosis of POAG. **Table S5.** Multivariable linear regression analyses of POAG genetic risk for intraocular pressure, refractive error, and corneal hysteresis. **Table S6.** Hazard ratio for incident POAG according to the degree of intraocular pressure, refractive error, and corneal hysteresis. **Table S7.** Concordance index of polygenic risk score and ocular parameters for incident glaucoma. **Table S8.** A summary table detailing the number of events and censored cases by each POAG genetic risk group.

**Additional file 2:** **Table S3.** Descriptions of disease phenotypes included in the PheWAS analyses. **Table S4.** Descriptions of disease phenotypes included in the PheWAS analyses for the diagnosis of POAG.

## Data Availability

The data that support the findings of this study are available from UK Biobank but restrictions may apply to the availability of these data, so they are not publicly available. However, the data are available from the authors with permission from the UK Biobank. The IGGC GWAS summary statistics data is available via GWAS Catalog, under the study accession identifier GCST90011767. The POAG PRS model and the PheWAS pipeline constructed in this study are available from the GitHub page (https://github.com/dokyoonkimlab/glaucoma-prs-phewas/).

## Abbreviations

BMI: Body mass index
CH: Corneal hysteresis
CI: Confidence interval
GWAS: Genome-wide association study
HR: Hazard ratio
ICD: International Classification of Diseases
IGGC: International Glaucoma Genetics Consortium
IOP: Intraocular pressure
OR: Odds ratio
PC: Principal component
PheWAS: Phenome-wide association study
POAG: Primary open angle glaucoma
PRS: Polygenic risk score
QC: Quality control
SD: Standard deviation
SE: Spherical equivalent
SNP: Single-nucleotide polymorphism

## References

[1] Zhang N, Wang J, Li Y (2021). Prevalence of primary open angle glaucoma in the last 20 years: a meta-analysis and systematic review. Sci Rep.

[2] Charlesworth J, Kramer PL, Dyer T (2010). The path to open-angle glaucoma gene discovery: endophenotypic status of intraocular pressure, cup-to-disc ratio, and central corneal thickness. Invest Ophthalmol Vis Sci.

[3] Polubriaginof FCG, Vanguri R, Quinnies K (2018). Disease heritability inferred from familial relationships reported in medical records. Cell.

[4] Wang K, Gaitsch H, Poon H (2017). Classification of common human diseases derived from shared genetic and environmental determinants. Nat Genet.

[5] Asefa NG, Neustaeter A, Jansonius NM (2019). Heritability of glaucoma and glaucoma-related endophenotypes: systematic review and meta-analysis. Surv Ophthalmol.

[6] Jonas JB, Aung T, Bourne RR (2017). Glaucoma. Lancet.

[7] Choi J, Kook MS (2015). Systemic and ocular hemodynamic risk factors in glaucoma. Biomed Res Int.

[8] Wu J, Hao J, Du Y (2022). The association between myopia and primary open-angle glaucoma: a systematic review and meta-analysis. Ophthalmic Res.

[9] Congdon NG, Broman AT, Bandeen-Roche K (2006). Central corneal thickness and corneal hysteresis associated with glaucoma damage. Am J Ophthalmol.

[10] Zimprich L, Diedrich J, Bleeker A (2020). Corneal hysteresis as a biomarker of glaucoma: current insights. Clin Ophthalmol.

[11] Jammal AA, Medeiros FA (2022). Corneal hysteresis: ready for prime time?. Curr Opin Ophthalmol.

[12] Murphy ML, Pokrovskaya O, Galligan M (2017). Corneal hysteresis in patients with glaucoma-like optic discs, ocular hypertension and glaucoma. BMC Ophthalmol.

[13] Gharahkhani P, Jorgenson E, Hysi P (2021). Genome-wide meta-analysis identifies 127 open-angle glaucoma loci with consistent effect across ancestries. Nat Commun.

[14] Craig JE, Han X, Qassim A (2020). Multitrait analysis of glaucoma identifies new risk loci and enables polygenic prediction of disease susceptibility and progression. Nat Genet.

[15] Wang Z, Wiggs JL, Aung T (2022). The genetic basis for adult onset glaucoma: recent advances and future directions. Prog Retin Eye Res.

[16] Siggs OM, Han X, Qassim A (2021). Association of monogenic and polygenic risk with the prevalence of open-angle glaucoma. JAMA Ophthalmol.

[17] Bycroft C, Freeman C, Petkova D (2018). The UK Biobank resource with deep phenotyping and genomic data. Nature.

[18] Conroy M, Sellors J, Effingham M (2019). The advantages of UK Biobank's open-access strategy for health research. J Intern Med.

[19] Howie BN, Donnelly P, Marchini J (2009). A flexible and accurate genotype imputation method for the next generation of genome-wide association studies. PLoS Genet.

[20] Fuchsberger C, Abecasis GR, Hinds DA (2015). minimac2: faster genotype imputation. Bioinformatics.

[21] Browning SR (2008). Missing data imputation and haplotype phase inference for genome-wide association studies. Hum Genet.

[22] Das S, Forer L, Schönherr S (2016). Next-generation genotype imputation service and methods. Nat Genet.

[23] Buniello A, MacArthur JAL, Cerezo M (2019). The NHGRI-EBI GWAS Catalog of published genome-wide association studies, targeted arrays and summary statistics 2019. Nucleic Acids Res.

[24] Ge T, Chen CY, Ni Y (2019). Polygenic prediction via Bayesian regression and continuous shrinkage priors. Nat Commun.

[25] Chang CC, Chow CC, Tellier LC (2015). Second-generation PLINK: rising to the challenge of larger and richer datasets. Gigascience.

[26] Denny JC, Ritchie MD, Basford MA (2010). PheWAS: demonstrating the feasibility of a phenome-wide scan to discover gene-disease associations. Bioinformatics.

[27] Denny JC, Bastarache L, Ritchie MD (2013). Systematic comparison of phenome-wide association study of electronic medical record data and genome-wide association study data. Nat Biotechnol.

[28] Zebardast N, Sekimitsu S, Wang J (2021). Characteristics of p.Gln368Ter myocilin variant and influence of polygenic risk on glaucoma penetrance in the UK Biobank. Ophthalmology..

[29] Zeleznik OA, Kang JH, Lasky-Su J (2023). Plasma metabolite profile for primary open-angle glaucoma in three US cohorts and the UK Biobank. Nat Commun.

[30] Chan MP, Grossi CM, Khawaja AP (2016). Associations with intraocular pressure in a large cohort: results from the UK Biobank. Ophthalmology.

[31] Zhang B, Shweikh Y, Khawaja AP (2019). Associations with corneal hysteresis in a population cohort: results from 96 010 UK Biobank participants. Ophthalmology.

[32] Khera AV, Emdin CA, Drake I (2016). Genetic risk, adherence to a healthy lifestyle, and coronary disease. N Engl J Med.

[33] Said MA, Verweij N, van der Harst P (2018). Associations of combined genetic and lifestyle risks with incident cardiovascular disease and diabetes in the UK Biobank Study. JAMA Cardiol.

[34] Siggs OM, Qassim A, Han X (2022). Association of high polygenic risk with visual field worsening despite treatment in early primary open-angle glaucoma. JAMA Ophthalmol.

[35] Zhou M, Wang W, Huang W (2014). Diabetes mellitus as a risk factor for open-angle glaucoma: a systematic review and meta-analysis. PLoS ONE.

[36] Zhao D, Cho J, Kim MH (2014). The association of blood pressure and primary open-angle glaucoma: a meta-analysis. Am J Ophthalmol.

[37] Delavar A, Radha Saseendrakumar B, Lee TC (2023). Associations between thyroid eye disease and glaucoma among those enrolled in the National Institutes of Health all of us research program. Ophthalmic Plast Reconstr Surg.

[38] Wang S, Liu Y, Zheng G (2017). Hypothyroidism as a risk factor for open angle glaucoma: a systematic review and meta-analysis. PLoS ONE.

[39] Cheong AJY, Wang SKX, Woon CY (2023). Obstructive sleep apnoea and glaucoma: a systematic review and meta-analysis. Eye (London)..

[40] Laville V, Kang JH, Cousins CC (2019). Genetic correlations between diabetes and glaucoma: an analysis of continuous and dichotomous phenotypes. Am J Ophthalmol.

[41] Kolli A, Sekimitsu S, Wang J (2023). Background polygenic risk modulates the association between glaucoma and cardiopulmonary diseases and measures: an analysis from the UK Biobank. Br J Ophthalmol.

[42] Chandrasekaran S, Cumming RG, Rochtchina E (2006). Associations between elevated intraocular pressure and glaucoma, use of glaucoma medications, and 5-year incident cataract: the Blue Mountains Eye Study. Ophthalmology.

[43] Chon B, Qiu M, Lin SC (2013). Myopia and glaucoma in the South Korean population. Invest Ophthalmol Vis Sci.

[44] Phelps CD, Burton TC (1977). Glaucoma and retinal detachment. Arch Ophthalmol.

[45] Han X, Gharahkhani P, Hamel AR (2023). Large-scale multitrait genome-wide association analyses identify hundreds of glaucoma risk loci. Nat Genet.

[46] Choquet H, Khawaja AP, Jiang C (2022). Association between myopic refractive error and primary open-angle glaucoma: a 2-sample mendelian randomization study. JAMA Ophthalmol.

[47] Sekimitsu S, Xiang D, Smith SL (2023). Deep ocular phenotyping across primary open-angle glaucoma. Genetic Burden JAMA Ophthalmol.

[48] Verma SS, Gudiseva HV, Chavali VRM, Salowe RJ, Bradford Y, Guare L (2024). A multi-cohort genome-wide association study in African ancestry individuals reveals risk loci for primary open-angle glaucoma. Cell.

[49] Gharahkhani P, Jorgenson E, Hysi P, Khawaja AP, Pendergrass S, Han X (2021). Genome-wide meta-analysis identifies 127 open-angle glaucoma loci with consistent effect across ancestries. Nat Commun.

[50] Choi SW, O'Reilly PF (2019). PRSice-2: Polygenic risk score software for biobank-scale data. Gigascience..

[51] Mak TSH, Porsch RM, Choi SW, Zhou X, Sham PC (2017). Polygenic scores via penalized regression on summary statistics. Genet Epidemiol.

[52] Privé F, Arbel J, Vilhjálmsson BJ (2021). LDpred2: better, faster, stronger. Bioinformatics..

[53] de Los CG, Vazquez AI, Fernando R, Klimentidis YC, Sorensen D (2013). Prediction of complex human traits using the genomic best linear unbiased predictor. PLoS Genet.

[54] Lloyd-Jones LR, Zeng J, Sidorenko J, Yengo L, Moser G, Kemper KE, Wang H, Zheng Z, Magi R, Esko T, Metspalu A, Wray NR, Goddard ME, Yang J, Visscher PM (2019). Improved polygenic prediction by Bayesian multiple regression on summary statistics. Nat Commun.

[55] Yang S, Zhou X (2020). Accurate and scalable construction of polygenic scores in large biobank data sets. Am J Hum Genet.

