# Supplementary material for: Polygenic risk score-based phenome-wide association for glaucoma and its impact on disease susceptibility in two large biobanks
Source: J Transl Med. 2024 Apr 15;22:355. doi: 10.1186/s12967-024-05152-4 (PMC11020996; doi:10.1186/s12967-024-05152-4)
Supplement: Supplementary file 1 — Additional file 1: Acknowledgements. Penn Medicine Biobank banner author list and contribution statements. Method S1. Detailed information on the genotype data quality control and imputation procedures. Figure S1. The flowchart showing participants included for analysis. Figure S2. Significance plot for all phenotypes for the diagnosis of primary open-angle glaucoma (POAG), grouped by disease categories. Figure S3. Log–log survival curves according to categories of polygenic risk score for primary open-angle glaucoma (POAG) (n = 73,548). Figure S4. Standardized cumulative incidence according to categories of polygenic risk score for primary open-angle glaucoma (POAG). Table S1. Descriptions of disease phenotypes included in the PheWAS analyses for POAG PRS. Table S2. Detailed definitions of glaucoma and comorbidities. Table S3. Demographic information for discovery and replication samples. Table S4. Descriptions of disease phenotypes included in the PheWAS analyses for the diagnosis of POAG. Table S5. Multivariable linear regression analyses of POAG genetic risk for intraocular pressure, refractive error, and corneal hysteresis. Table S6. Hazard ratio for incident POAG according to the degree of intraocular pressure, refractive error, and corneal hysteresis. Table S7. Concordance index of polygenic risk score and ocular parameters for incident glaucoma. Table S8. A summary table detailing the number of events and censored cases by each POAG genetic risk group. [file 12967_2024_5152_MOESM1_ESM.pdf]

Additional file 1.

Polygenic Risk Score-based Phenome-wide Association for Glaucoma and Its Impact on Disease Susceptibility in Two Large Biobanks

Appendix

Acknowledgements. Penn Medicine Biobank banner author list and contribution statements.

Method S1. Detailed information on the genotype data quality control and imputation procedures.

Figure S1. Flowchart showing participants included for analysis.
Figure S2. Significance plot for all phenotypes for the diagnosis of primary open-angle glaucoma (POAG), grouped by disease categories.
Figure S3. Log-log survival curves according to categories of polygenic risk score for primary open-angle glaucoma (POAG) (n=73,548).
Figure S4. Standardized cumulative incidence according to categories of polygenic risk score for primary open-angle glaucoma (POAG).

Table S1. Descriptions of disease phenotypes included in the PheWAS analyses for POAG PRS.
Table S2. Detailed definitions of glaucoma and comorbidities.
Table S3. Demographic information for discovery and replication samples.
Table S4. Descriptions of disease phenotypes included in the PheWAS analyses for the diagnosis of POAG.
Table S5. Multivariable linear regression analyses of POAG genetic risk for intraocular pressure, refractive error, and corneal hysteresis.
Table S6. Hazard ratio for incident POAG according to the degree of intraocular pressure, refractive error, and corneal hysteresis.
Table S7. Concordance index of polygenic risk score and ocular parameters for incident glaucoma.
Table S8. A summary table detailing the number of events and censored cases by each POAG genetic risk group.

Acknowledgements. Penn Medicine Biobank banner author list and contribution statements.

PMBB Leadership Team

Daniel J. Rader, M.D., Marylyn D. Ritchie, Ph.D.

Contribution: All authors contributed to securing funding, study design and oversight. All authors reviewed the final version of the manuscript.

Patient Recruitment and Regulatory Oversight

JoEllen Weaver, Nawar Naseer, Ph.D., M.P.H., Giorgio Sirugo, M.D., P.h.D., Afiya Poindexter, Yi-An Ko, Ph.D., Kyle P. Nerz

Contributions: JW manages patient recruitment and regulatory oversight of study. NN manages participant engagement and assists with regulatory oversight, and researcher access. GS assists with researcher access. AP, YK, KPN perform recruitment and enrollment of study participants.

Lab Operations

JoEllen Weaver, Meghan Livingstone, Fred Vadivieso, Stephanie DerOhannessian, Teo Tran, Julia Stephanowski, Salma Santos, Ned Haubein, P.h.D., Joseph Dunn

Contribution: JW, ML, FV, SD conduct oversight of lab operations. ML, FV, AK, SD, TT, JS, SS perform sample processing. NH, JD are responsible for sample tracking and the laboratory information management system.

Clinical Informatics

Anurag Verma, Ph.D., Colleen Morse Kripke, M.S. DPT, MSA, Marjorie Risman, M.S., Renae Judy, B.S., Colin Wollack, M.S.

Contribution: All authors contributed to the development and validation of clinical phenotypes used to identify study subjects and (when applicable) controls.

Genome Informatics

Anurag Verma Ph.D., Shefali S. Verma, Ph.D., Scott Damrauer, M.D., Yuki Bradford, M.S., Scott Dudek, M.S., Theodore Drivas, M.D., Ph.D.,

Contribution: AV, SSV, and SD are responsible for the analysis, design, and infrastructure needed to quality control genotype and exome data. YB performs the analysis. TD and AV provide variant and gene annotations and their functional interpretation of variants.

Method S1. Detailed information on the genotype data quality control and imputation procedures.

UK Biobank

The UK Biobank samples (version 3; March 2018) were genotyped using either the Affymetrix UK BiLEVE Axiom array or the Affymetrix UK Biobank Axiom array; these encompass >800,000 genotyped SNPs, 95% of which are shared between the two platforms. Imputation via IMPUTE2 was conducted by UK Biobank researchers using the merged 1000 Genomes Project panel and UK 10K panel. After imputation, variant-level quality control (QC) was performed by filtering SNPs on two criteria: i) minor allele frequency <0.01 and ii) imputation quality score (INFO) <0.3. A total of 9,505,768 imputed autosomal SNPs passed the QC criteria. Sample-level QC was performed by excluding samples on the basis of i) participants identified as not of 'white-British' ancestry according to either self-report or principal component (PC) analysis of genetic ancestry, ii) mismatched sex, or iii) having second-degree or closer relatives also in the Biobank. After exclusion, 377,852 white-British participants were determined eligible for the genetic analyses.

Penn Medicine Biobank

The Penn Medicine Biobank consists of 43,623 unique samples that underwent genotyping with the GSA genotyping array (Illumina, SD, USA). For the Penn Medicine Biobank dataset, we performed genotype imputation using Eagle and Minimac software on the TOPMed Imputation Server. Imputation was applied over all autosomes, with TOPMed version R2 on the GRCh38 reference panel. Variant-level QC was performed by filtering SNPs on three criteria: i) minor allele frequency <0.01, ii) marker call rate <0.95, and iii) INFO <0.2. Sample-level QC was performed by excluding samples on the basis of i) participants identified as not of 'Non-Hispanic-White' ancestry according to either self-report or PC analysis of genetic ancestry, ii) mismatched sex, or iii) having second-degree or closer relatives also in the Biobank. After exclusion, 27,933 White-European participants were deemed eligible for the replication analyses.

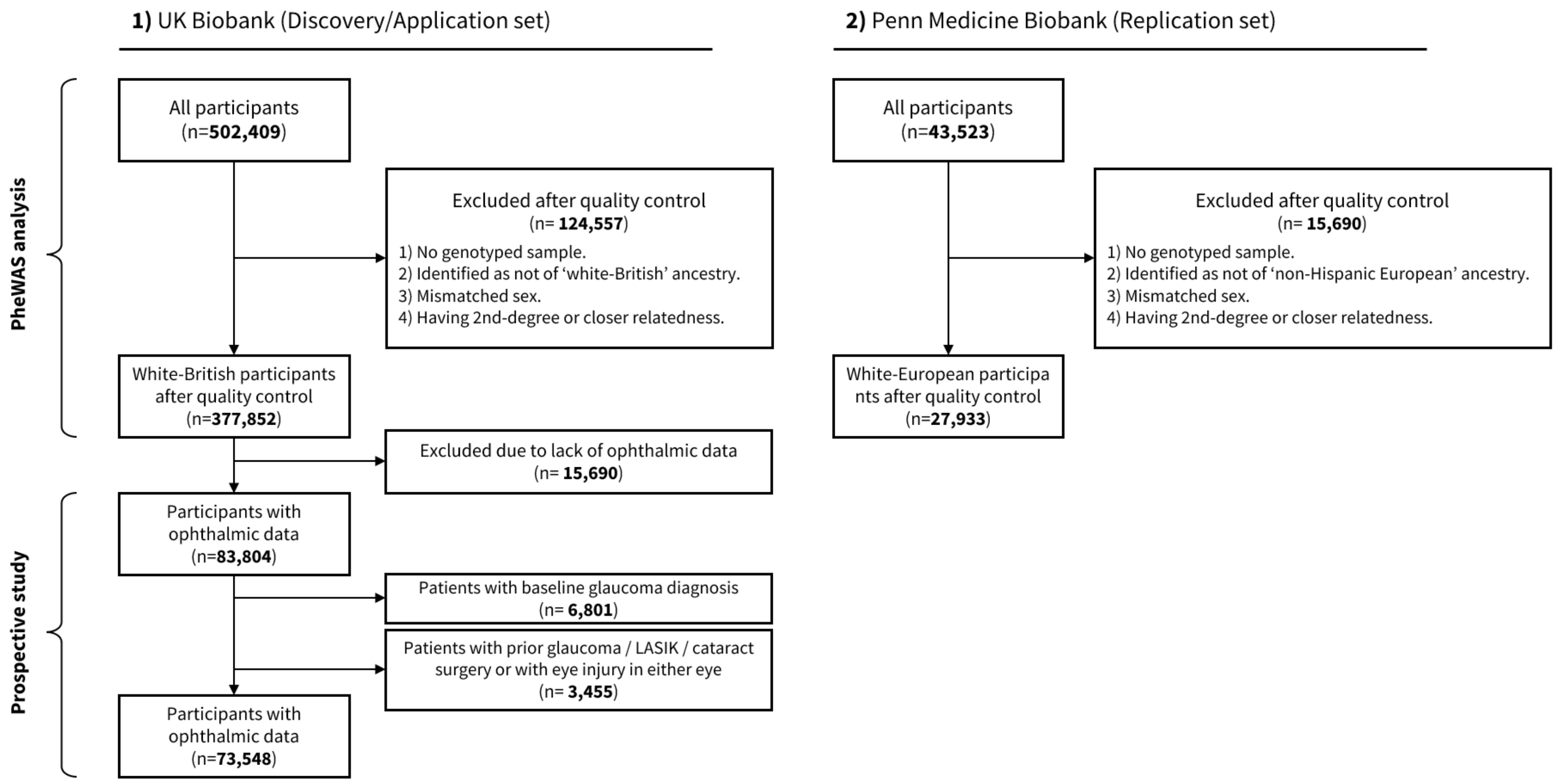

Figure S1. Flowchart showing participants included for analysis.

PheWAS, phenome-wide association study.

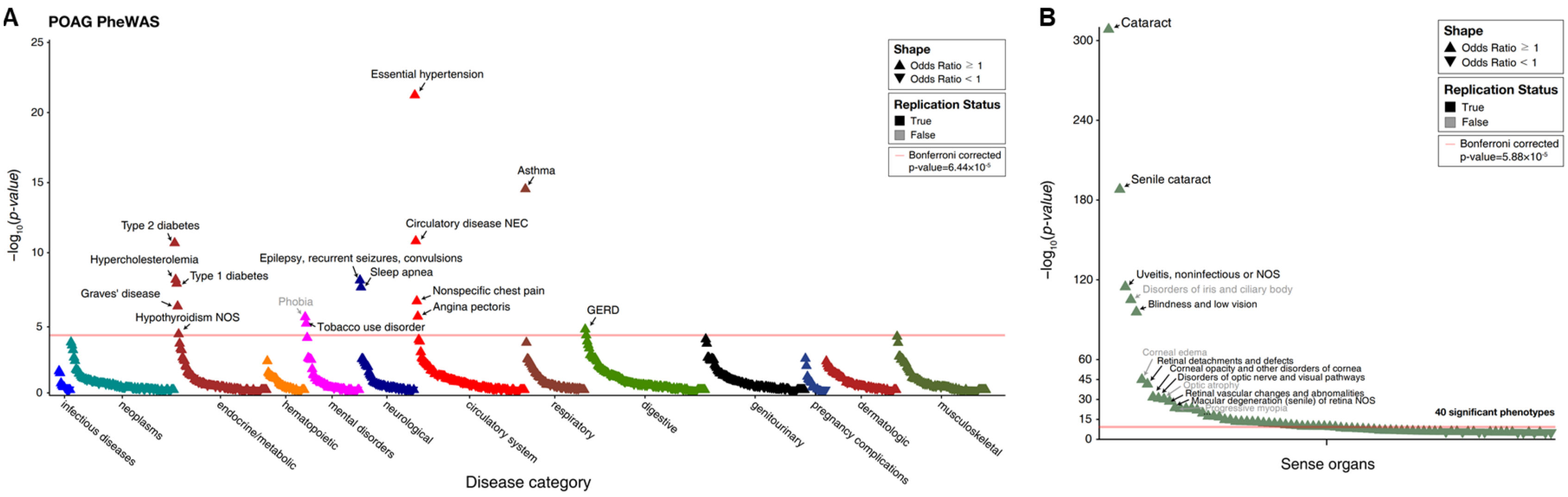

Figure S2. Significance plot for all phenotypes for the diagnosis of primary open angle glaucoma (POAG), grouped by disease categories. Significance plot for all phenotypes for the diagnosis of POAG, grouped by disease categories. PheCodes are organized and plotted by disease categories other than sensory organ system **(A)** and significance plot for sensory organ system **(B)** on the *x*-axis, and the *y*-axis represents the $-\log_{10}$ of uncorrected *P* values of two-sided test for linear regression between POAG and each of the phenotype. Each point represents a single PheCode, and the color indicates their corresponding categories. Among PheCodes that reached phenome-wide significance (horizontal red line, $P < 6.44\times10^{-5}$ and $P < 5.88\times10^{-5}$ for disease categories other than sensory organ system and for sensory organ system, respectively), the representative significant associations are annotated. The PheCodes that displayed significant associations in both the UK Biobank and the Penn Medicine Biobank are represented by black letters in the figure, while those exhibiting significant associations only in the UK Biobank are indicated by gray letters. The direction of each arrowhead corresponds to increased risk (up) or decreased risk (down). NOS, not otherwise specified; NEC, not elsewhere classifiable. The majority of significant associations were observed for diseases in the circulatory system, including essential hypertension ($P = 2.96 \times 10^{-25}$), circulatory disease ($P = 2.96 \times 10^{-25}$), non-specific chest pain ($P = 2.85 \times 10^{-7}$), and angina pectoris ($P = 3.52 \times 10^{-6}$), as well as the endocrine/metabolic system, encompassing type 2 and type 1 diabetes ($P = 2.03 \times 10^{-11}$ and $P = 1.57 \times 10^{-8}$), hypercholesterolemia ($P = 8.34 \times 10^{-9}$), Grave's disease ($P = 6.60 \times 10^{-7}$), and hypothyroidism ($P = 6.41 \times 10^{-5}$). Additionally, a significant association was noted between POAG diagnosis and conditions such as asthma ($P = 2.87 \times 10^{-15}$), sleep apnea ($P = 2.92 \times 10^{-8}$) and gastroesophageal reflux disease ($P = 7.68 \times 10^{-5}$). Most of the disease phenotypes that exhibited significant associations in the discovery set demonstrated consistent directional effects with statistical significance in the replication set (**Table S4**). Within the disease category of the sensory organ system, 40 significant ocular phenotypes were found to be strongly linked to the diagnosis of POAG, including such as cataract, uveitis, blindness or low vision, retinal detachment, and myopia.

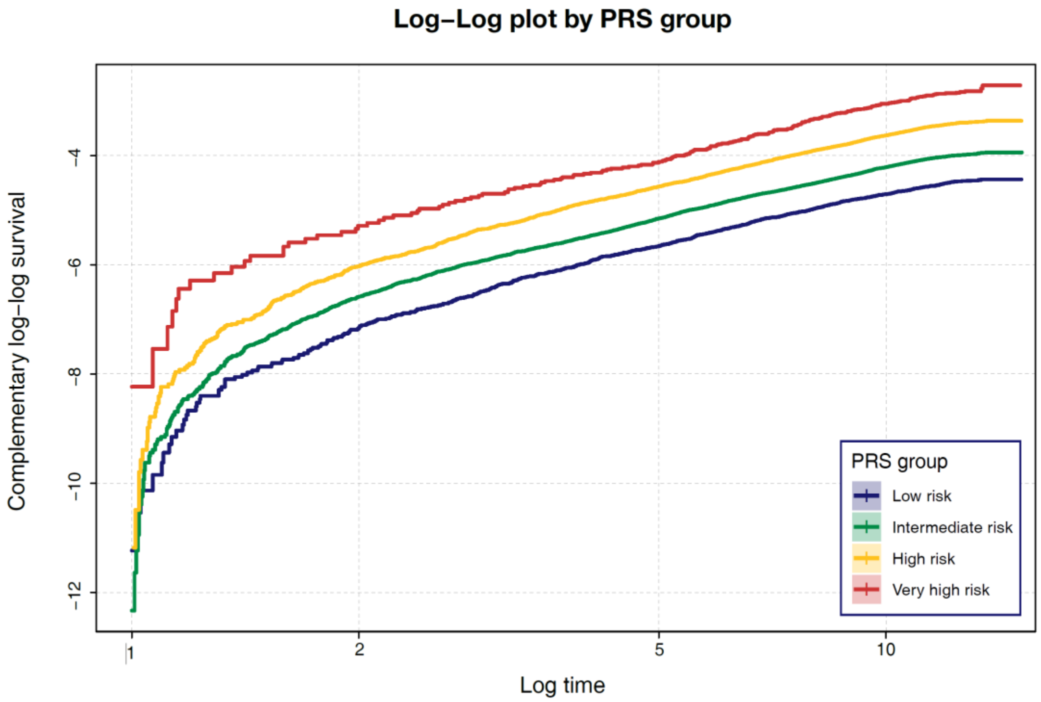

Figure S3. Log-log survival curves according to categories of a polygenic risk score for primary open-angle glaucoma (POAG) (n=73,548). In the log-log plots, each plot illustrates the survival probabilities over time for different genetic risk groups and confirmed the parallel trends across groups, indicating the validity of the proportional hazards assumption.

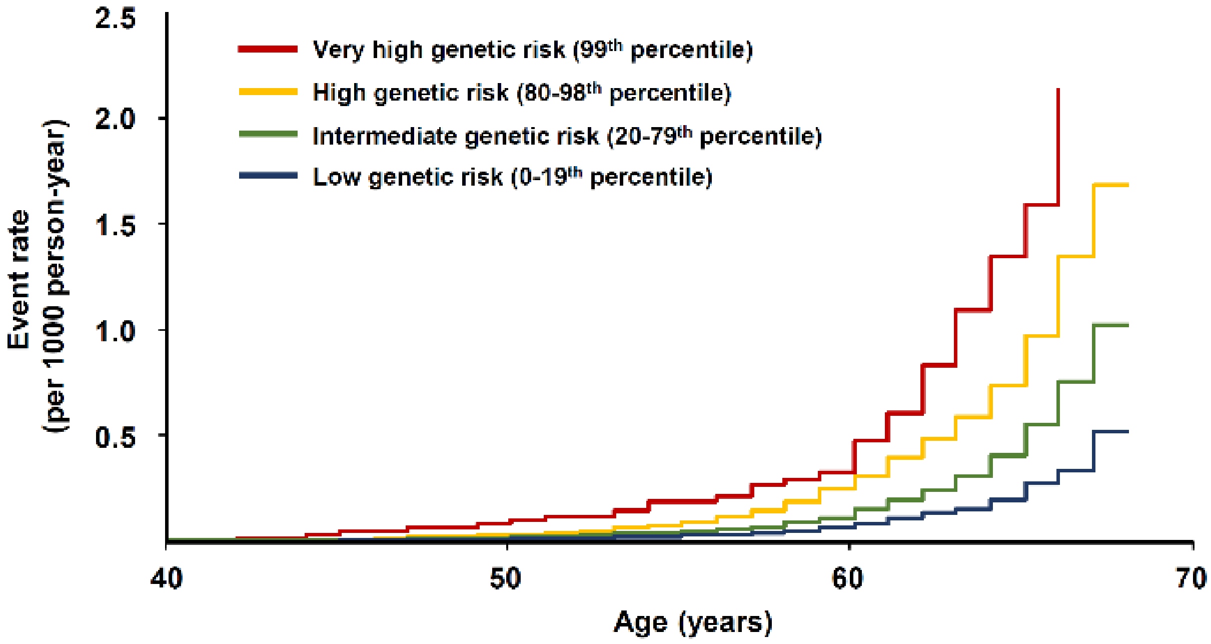

Figure S4. Standardized cumulative incidence according to categories of polygenic risk score for primary open angle glaucoma (POAG) (n=73,548).

Table S1. Demographic information for discovery and replication samples.

No.	**Discovery Sample: UK Biobank (UK)** (n=377,852)	**Replication Sample: Penn Medicine Biobank (USA)** (n=27,933)
Age, mean (SD), y	57·5 ± 7·9	57·4 ± 16·2
Sex, No. (%)		
Male	174,803 (46·3%)	15,318 (54·8%)
Female	203,049 (53·7%)	12,615 (45·2%)
Ancestry	White-British	White-European (non-Hispanic)
Education years, mean (SD), y	13·8 ± 5·1	n/a
Number in household, mean (SD)	2·4 ± 1·2	n/a
Townsend deprivation index, mean (SD)	-1·6 ± 2·9	n/a
Average total household income before tax		n/a
Less than £18,000	71,985 (22·1%)	
1,8000 to 30,999£	83,995 (25·8%)	
31,000 to 51,999£	85,831 (26·4%)	
52,000 to 100,000£	66,379 (20·4%)	
Greater than 100,000£	17,023 (5·2%)	
Body mass index, mean (SD), kg/m^2	27·4 ± 4·8	28·4 ± 6·3
Body mass index, No. (%)		
≥30 kg/m^2	91,462 (24·3%)	9,038 (33·6%)
<30 kg/m^2	285,255 (75·7%)	17,893 (66·4%)
Waist circumference, mean (SD), cm	90·4 ± 13·5	n/a
Systolic blood pressure, mean (SD), mm Hg	140·3 ± 19·7	n/a
Diastolic blood pressure, mean (SD), mm Hg	82·3 ± 10·7	76·0 ± 12·5
HbA1c, mean (SD), (%)	5·4 ± 0·6	6·2 ± 2·1
Estimated GFR, mean (SD), $ml/min/1.73m^2$	78·6 ± 14·2	81·1 ± 23·5
Total cholesterol, mean (SD), mmol/L	5·72 ± 1·15	4·70 ± 1·23
Triglyceride, mean (SD), mmol/L	1·76 ± 1·02	1·53 ± 1·49
HDL-cholesterol, mean (SD), mmol/L	1·45 ± 0·38	1·35 ± 0·46
LDL-cholesterol, mean (SD), mmol/L	3·57 ± 0·87	2·69 ± 0·97
Smoking history, No. (%)		
Never	204,979 (54·4%)	13,793 (51·0%)
Ever	171,635 (45·6%)	13,257 (49·0%)

SD, standard deviation; HDL, High-density lipoprotein; LDL, Low-density lipoprotein; eGFR, Estimated Glomerular Filtration Rate.

Table S2. Detailed definitions of glaucoma and comorbidities.

Disease	Path	Field ID	Code
Baseline glaucoma	Verbal interview	Non-cancer illness, self-report (20002)	Glaucoma (1277)
	First occurrence before enrollment	First reported of glaucoma (131186, 131187)	H40.x
		First reported of glaucoma in disease classified elsewhere (131188, 131189)	H42.x
	Hospital data before enrollment	Summary Diagnosis (ICD10, 41270)	H40.1, H40.8, H40.9
		Summary Diagnosis (ICD9, 41271)	36511, 3659
Glaucoma event during follow-up	First occurrence after enrollment	First reported of glaucoma (131186, 131187)	H40.x
		First reported of glaucoma in disease classified elsewhere (131188, 131189)	H42.x (excluded)
	Hospital data after enrollment	Summary Diagnosis (ICD10, 41270)	H40.1, H40.8, H40.9
		Summary Diagnosis (ICD9, 41271)	36511, 3659
Baseline dyslipidemia	Verbal interview	Non-cancer illness, self-report (20002)	High cholesterol (1473)
	First occurrence before enrollment	First reported of disorders of lipoprotein metabolism and other lipidemia (130815, 130816)	E78.x
	Medication	Medication for cholesterol, blood pressure or diabetes (6177)	Cholesterol lowering medication
Baseline hypertension	Verbal interview	Non-cancer illness, self-report (20002)	1065, 1072
	Touchscreen	Vascular/heart problems diagnosed by doctor (6150)	High blood pressure
	First occurrence before enrollment	First reported of essential hypertension (131286, 131287)	I10.x
		First reported of hypertensive heart disease (131288, 131289)	I11.x
		First reported of hypertensive renal disease (131290, 131291)	I12.x
		First reported of hypertensive heart and renal disease (131292, 131293)	I13.x
		First reported of secondary hypertension (131294, 131295)	I15.x
	Medication	Medication for cholesterol, blood pressure or diabetes (6177)	Blood pressure medication
Baseline type 2 diabetes mellitus	Verbal interview	Non-cancer illness, self-report (20002)	Diabetes (1220)
			Type 2 diabetes (1223)
	Touchscreen	Diabetes diagnosed by doctor (2443)	Yes
	First occurrence before enrollment	First reported of non-insulin-dependent diabetes mellitus (130708, 130709)	E11.x
		First reported of unspecified diabetes mellitus (130714, 130715)	E14.x
	Medication	Treatment/medication code (20003)	Insulin (1140883066)
			Metformin (1140884600, 1141189090)

		Sulfonylurea (1141152590, 1140874744, 1140874718, 1141156984)<br>Acarbose (1140868902)<br>Thiazolidinedione (1141171646)<br>Meglitinide (1141168660, 1141173882)
HbA1c at baseline	Glycated hemoglobin (HbA1c) (30750)	≥6.5%
Verbal interview for exclusion type 1 diabetes	Non-cancer illness, self-report (20002)	Type 1 diabetes (1222)
First occurrence for exclusion type 1 diabetes	First reported of insulin-dependent diabetes mellitus (130706, 130707)	E10.x

Table S3. Descriptions of disease phenotypes included in the PheWAS analyses for POAG PRS.

※ The Supplemental Table is provided as a separate Excel file (Additional file 2).

Table S4. Descriptions of disease phenotypes included in the PheWAS analyses for the diagnosis of POAG.

※ The Supplemental Table is provided as a separate Excel file (Additional file 2).

Table S5. Multivariable linear regression analyses of POAG genetic risk for intraocular pressure, refractive error, and corneal hysteresis (n=73,548).

	IOP_g (mm Hg)								
	Crude			Model 1			Model 2		
	Beta	95% CI	*P* value	Beta	95% CI	*P* value	Beta	95% CI	*P* value
Glaucoma PRS mean (SD)	3.560	3.370-3.740	< 0.0001	3.580	3.390- 3.760	<0.0001	3.530	3.320 – 3.740	<0.0001
	SE (diopter)								
	Crude			Model 1			Model 2		
	Beta	95% CI	*P* value	Beta	95% CI	*P* value	Beta	95% CI	*P* value
Glaucoma PRS mean (SD)	-0.730	-0.862– -0.599	< 0.0001	-0.705	-0.835– -0.576	<0.0001	-0.635	-0.782– -0.487	<0.0001
	CH (mm Hg)								
	Crude			Model 1			Model 2		
	Beta	95% CI	*P* value	Beta	95% CI	*P* value	Beta	95% CI	*P* value
Glaucoma PRS mean (SD)	-0.433	-0.551– -0.315	< 0.0001	-0.439	-0.556– -0.321	<0.0001	-0.393	-0.529– -0.258	<0.0001

POAG, primary open angle glaucoma; PRS, polygenic risk score; SE, spherical equivalent; CH, corneal hysteresis; IOP_g, Goldmann-correlated intraocular pressure; SD, standard deviation; CI, confidence interval.

Model 1: Age + sex + genotyping array + first ten principal components of ancestry.

Model 2: Model 1+ BMI + Income+ Smoking + systolic blood pressure + diastolic blood pressure + LDL cholesterol + HbA1c + hypertension

Table S6. Hazard ratio for incident POAG according to the degree of intraocular pressure, refractive error, and corneal hysteresis (n=73,548).

	Crude		Model 1		Model 2	
	HR (95% CI)	P value	HR (95% CI)	P value	HR (95% CI)	P value
IOP, mm Hg	1.10 (1.10-1.11)	< 0.001	1.10 (1.10-1.11)	< 0.001	1.10 (1.09-1.11)	< 0.001
SE, diopter	0.98 (0.96-0.99)	< 0.001	0.95 (0.93-0.96)	< 0.001	0.94 (0.92-0.96)	< 0.001
CH, mm Hg	0.92 (0.89-0.94)	< 0.001	0.94 (0.92-0.97)	< 0.001	0.96 (0.94-0.99)	< 0.001

POAG, primary open angle glaucoma; IOP; intraocular pressure, SE; spherical equivalent, CH; corneal hysteresis; ; HR, Hazard ratio; CI, confidence interval;

Model 1: Age + sex + genotyping array + first ten principal components of ancestry.

Model 2: Model 1 + BMI + smoking status + systolic blood pressure + diastolic blood pressure + LDL cholesterol + HbA1c + Hypertension + + use of anti-glaucoma eyedrops

Table S7. Concordance index of polygenic risk score and ocular parameters for incident glaucoma.

Parameters	Concordance index (95% CI)
Glaucoma PRS	0.628 (0.618-0.643)
SE	0.497 (0.484-0.512)
CH	0.561 (0.547-0.577)
IOP	0.743 (0.727-0.757)
Glaucoma PRS + SE	0.628 (0.611-0.644)
Glaucoma PRS + CH	0.638 (0.625-0.654)
Glaucoma PRS + IOP	0.741 (0.730-0.752)
Glaucoma PRS + SE + CH + IOP	0.744 (0.733-0.757)

PRS, polygenic risk score; SE, spherical equivalent; CH, corneal hysteresis; IOP, intraocular pressure; CI, confidence interval.

Table S8. A summary table detailing the number of events and censored cases by each POAG genetic risk group.

Glaucoma PRS group	Event number	Censored number
Low risk	889	74,683
Intermediate risk	4,298	222,411
High risk	2,444	69,348
Very high risk	228	3,551
	7,859	369,933

PRS, polygenic risk score.
